# Supplementary material for: End Sequence Analysis Toolkit (ESAT) expands the extractable information from single-cell RNA-seq data
Source: Genome Res. 2016 Oct;26(10):1397–410. doi: 10.1101/gr.207902.116 (PMC5052061; doi:10.1101/gr.207902.116)
Supplement: Supplemental Material [file supp_gr.207902.116_Supp_Materials.tar.gz › ESAT-esat_v0.1/doc/html/functions_f.html]

ESAT: Class Members


ESAT


- Main Page
- Related Pages
- Packages
- Classes
- Files

- Class List
- Class Index
- Class Hierarchy
- Class Members

- All
- Functions
- Variables
- Enumerator

- a
- b
- c
- d
- e
- f
- g
- h
- i
- j
- k
- l
- m
- n
- o
- p
- q
- r
- s
- t
- u
- v
- w
- z


All Classes Namespaces Files Functions Variables Enumerator Pages

Here is a list of all class members with links to the classes they belong to:

### - f -

- F()
  : broad.core.math.ANOVA
- factorial()
  : broad.core.math.MathUtil
- FASTA()
  : jaligner.formats.FASTA
- FastaExtendPeaks()
  : broad.pda.seq.clip.FastaExtendPeaks
- FastaQualityIndex()
  : broad.core.sequence.FastaQualityIndex
- FastaSequenceIO()
  : broad.core.sequence.FastaSequenceIO
- FastqLibraryStats()
  : broad.pda.countreads.FastqLibraryStats
- FastqParser()
  : broad.pda.seq.fastq.FastqParser
- FastqSequence()
  : broad.pda.seq.fastq.FastqSequence
- FastqSplitterByBarcode()
  : broad.pda.seq.fastq.FastqSplitterByBarcode
- FDR()
  : broad.core.math.ComputeFDR
  , broad.core.math.Statistics
- FDRCorrect()
  : broad.core.math.Statistics
- FDRDistribution()
  : broad.core.math.FDRDistribution
- FDRExact()
  : broad.core.math.ComputeFDR
- FDRExactPrint()
  : broad.core.math.ComputeFDR
- FeatureSequenceAlignment()
  : umms.core.alignment.FeatureSequenceAlignment
- fileSize
  : broad.core.sequence.AbstractFastaParser
- filter()
  : broad.core.util.CollapseByIntersection
- FILTER\_REJECT\_DIR
  : broad.pda.seq.protection.MultiSampleScanPeakCaller
- filterByOverlap()
  : broad.core.annotation.AnnotationReader< T extends GenomicAnnotation >
  , broad.pda.annotation.BEDFileParser
- filterByOverlapAndOrientation()
  : broad.core.annotation.AnnotationReader< T extends GenomicAnnotation >
- filterByOverlapOfLastExon()
  : broad.core.annotation.AnnotationReader< T extends GenomicAnnotation >
- filterByOverlapOfLastExonOrAfterLastExon()
  : broad.core.annotation.AnnotationReader< T extends GenomicAnnotation >
- filterByScore()
  : broad.core.annotation.AnnotationReader< T extends GenomicAnnotation >
- FilteredIterator()
  : umms.core.model.AlignmentModel.FilteredIterator
  , umms.core.model.JCSAlignmentModel.FilteredIterator
- FilterGCTFile()
  : broad.pda.geneexpression.FilterGCTFile
- filterGenesByExpression()
  : broad.pda.seq.segmentation.ContinuousDataAlignmentModel
- filterInvariantGenes()
  : broad.core.datastructures.MatrixWithHeaders
- FilterMatrixByMinCutoff()
  : broad.pda.geneexpression.agilent.FilterMatrixByMinCutoff
- filterPaths()
  : broad.pda.seq.segmentation.ContinuousDataAlignmentModel
- filterValuesLargerThanUsingColumn()
  : broad.core.datastructures.MatrixWithHeaders
- filterValuesRangeUsingColumn()
  : broad.core.datastructures.MatrixWithHeaders
- filterValuesSmallerThanUsingColumn()
  : broad.core.datastructures.MatrixWithHeaders
- filterWindows()
  : broad.pda.seq.protection.TwoSampleScanSkellamPeakCaller
- find()
  : broad.core.annotation.BasicGenomicAnnotationReader
  , broad.core.datastructures.IntervalTree< V >
- findAllORFs()
  : umms.core.annotation.Gene
- findAllStartCodons()
  : umms.core.annotation.Gene
- findAnnotationFiles()
  : broad.pda.enrichment.EnrichmentUtils
- findByIndex()
  : broad.core.datastructures.IntervalTree< V >
- findClosest()
  : broad.core.annotation.AnnotationReader< T extends GenomicAnnotation >
- findClosestForAll()
  : broad.core.annotation.AnnotationReader< T extends GenomicAnnotation >
- findClosestNonOverlapping()
  : broad.core.annotation.AnnotationReader< T extends GenomicAnnotation >
- findClosestNonoverlappingForAll()
  : broad.core.annotation.AnnotationReader< T extends GenomicAnnotation >
- findCompatibleGenes()
  : broad.pda.gene.GeneWithIsoforms
- FindDiffrentialEnrichedRegions()
  : broad.pda.seq.chip.FindDiffrentialEnrichedRegions
- findEdge()
  : broad.pda.seq.graph.ChromosomeWithBubblesJGraphT
- FindEnrichedDepletedRegions()
  : broad.pda.rap.misc.FindEnrichedDepletedRegions
- findLongestORF()
  : umms.core.annotation.Gene
- FindProbesForGeneSet()
  : broad.pda.geneexpression.agilent.FindProbesForGeneSet
- findReachableTargetVertices()
  : broad.pda.seq.graph.ChromosomeWithBubblesJGraphT
- findUngappedSequenceChunks()
  : broad.core.sequence.Sequence
- finish()
  : broad.pda.seq.rap.rna.CollectRnaSeqMetrics
- finishedRegion()
  : umms.core.model.score.WindowProcessor< S extends WindowScore >.AbstractProcessor< S extends WindowScore >
  , umms.core.model.score.WindowProcessor< S extends WindowScore >
- firstField()
  : broad.core.parser.StringParser
- firstReadTranscriptionStrand()
  : broad.pda.seq.clip.SampleData
  , broad.pda.seq.protection.SampleData
- FisherCombinedProbabilityTest()
  : broad.core.math.FisherCombinedProbabilityTest
- fitOmega()
  : broad.core.siphy.EvolutionaryModel
  , broad.core.siphy.tools.conservation.EstimateOmegaPerExon
- fitPI()
  : broad.core.siphy.EvolutionaryModel
  , broad.core.siphy.TreeScaler
- flatWeight()
  : broad.core.motif.PositionWeightColumn
- flatWeightMatrix()
  : broad.core.motif.PositionWeightMatrix
- floor()
  : broad.core.datastructures.MatrixWithHeaders
- FokI
  : broad.core.primer3.RestrictionEnzyme
- fold()
  : broad.core.math.Statistics
- FoldSignificance()
  : broad.pda.differentialExpression.FoldSignificance
- format()
  : jaligner.formats.CLUSTAL
  , jaligner.formats.FASTA
  , jaligner.formats.Format
  , jaligner.formats.Pair
- Fp()
  : broad.core.math.ScanStatistics
- FpWithBreaking()
  : broad.core.math.ScanStatistics
- FragmentAlignment()
  : umms.core.alignment.FragmentAlignment
- fromFullInfoString()
  : umms.core.annotation.BasicAnnotation
- fromString()
  : umms.core.annotation.Annotation.Strand
- FullyContainedFilter()
  : umms.core.annotation.filter.FullyContainedFilter
- fullyContainedReads
  : broad.pda.seq.clip.SampleData
  , broad.pda.seq.protection.SampleData
  , broad.pda.seq.protection.WindowScores
- fullyContains()
  : broad.core.sequence.SequenceRegion
  , umms.core.annotation.AbstractAnnotation
  , umms.core.annotation.Annotation
- functionalAnnotation()
  : broad.pda.gene.functionalAnnotation
- FwdIterator()
  : broad.core.datastructures.IntervalTree< V >.FwdIterator
- FWERDistribution()
  : broad.pda.differentialExpression.FWERDistribution


---

Generated on Fri Aug 15 2014 15:11:16 for ESAT by  

 1.8.7
